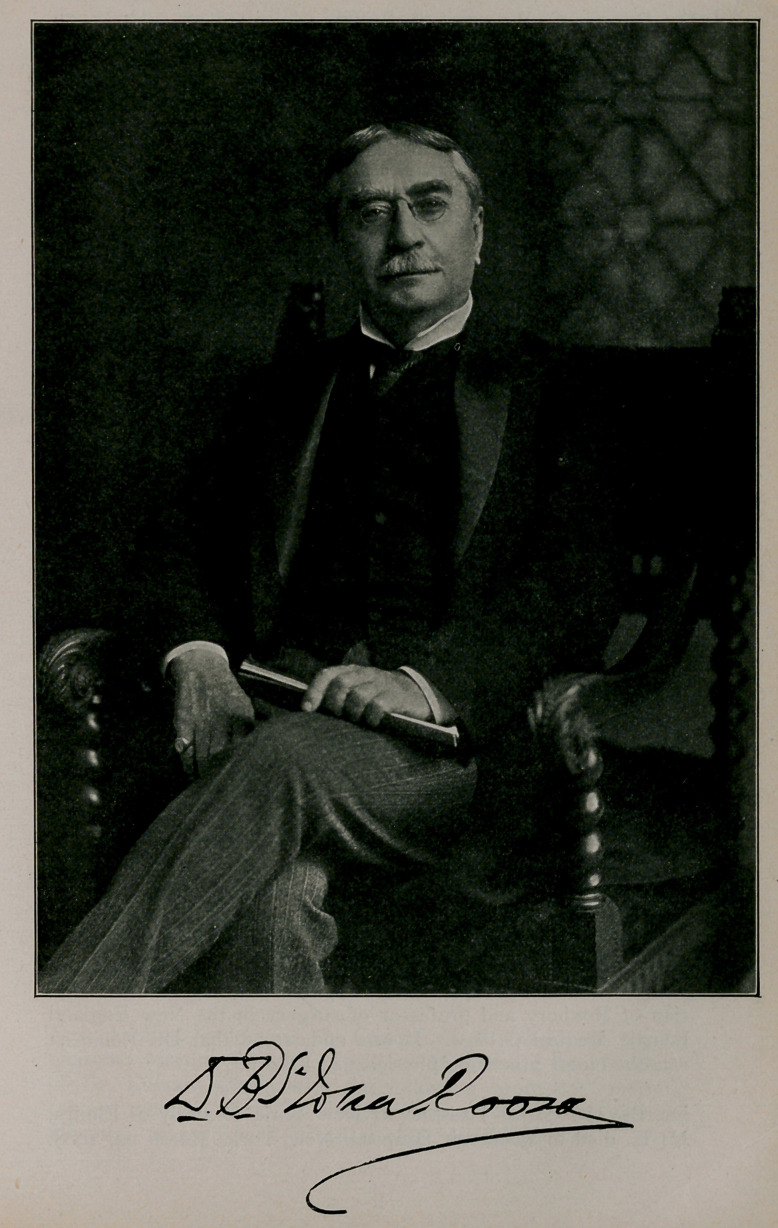# Personal

**Published:** 1908-05

**Authors:** 


### PERSONAL.

DR. L. S. MCMURTRY, of Louisville, by invitation, delivered the principal address on the occasion of the celebration of the twenty-fifth anniversary of the Washington Obstetrical and Gynecological Society, which occurred March 6, 1908. We hope to publish the address in a subsequent issue. It dealt with the accomplishments and triumphs of gynecology during the past quarter of a century. Dr. McMurtry was the guest of the society during his visit in Washington.

---

DR. JOHN E. CANNADAY, of Wheeling, West Va., has removed to 1012 Virginia Street, Charleston, W. Va.

Dr. Eli H .Long, professor of materia medica and therapeutics at the University of Buffalo, was elected president of the Association of American Medical Colleges at its annual meeting held at Cleveland, March 16, 1908.

---

Dr. Herbert U. Williams, professor of pathology and bacteriology at the University of Buffalo, was elected treasurer of the American Association of Pathologists and Bacteriologists at its annual meeting held at Ann Arbor, Mich., April 17, 1908.

---

Dr. Stephen Yates Howell, of Buffalo, who has been abroad for three months returned home April 24, 1908, and resumed his surgical practice. During his visit to England he spent some time in London in anatomical study and surgical work at King's College and other institutions. He also stayed several weeks at Leeds with Mr. Moynihan in witnessing his special work, and in doing the special operations for which Mr. Moynihan is so famous.

---

Dr. William H. Billings of Buffalo, announces his removal from 271 Caroline Street to 1272 Main Street. He limits his practice to flat foot and other deformities of the feet. Telephones: Bell, Bryant 981; Frontier 1434. Hours: 9 to 10, 1 to 3. Evenings by appointment.

---

Dr. Robert B. Blanchard, of Jamestown, N. Y., has been reappointed city physician of that town, the common council having confirmed him April 20, 1908, first voting down the mayor's appointment of another physician. Dr. Blanchard graduated from the medical department of the University of Buffalo in 1906.

---

Dr. John O. Aldrich, of Bath, N. Y., has been elected president of the board of health of that village. Dr. Harry Wynkoop was elected secretary and Dr. Deyo Matthewson was chosen health officer. The board passed a resolution, at a recent meeting, prohibiting spitting on the streets.

---

Passed Assistant Surgeon C. F. Ely, United States navy, who has been stationed in Buffalo for nearly two years, has been ordered to the training ship Hartford to assume charge of the hospital corps on board that vessel. The Hartford is at Norfolk, Va., and is used in training the midshipmen from the naval academy at Annapolis.

---

Dr. Herbert M. Hill, of Buffalo, professor of chemistry at the University, has occupied his new home at the corner of Bryant Street and Norwood Avenue.

Born April 4, 1838, Died March 8, 1908.

Dr. Dewitt G. Wilcox, of Buffalo, delivered an address on Obstetric Surgery at the meeting of the Southern Tier Homeopathic Medical Association at its meeting held in Elmira, N. Y., April 21, 1908.

---

Dr. William Warren Potter, of Buffalo, President of the New York State Board of Medical Examiners, was commissioned by Governor Hughes to represent the State of New York at the Fourth Annual Conference of the Council on Medical Education of the American Medical Association held at Chicago, April 13, 1908. Dr. Potter also represented the state examining board at the conference. The proceedings are reported elsewhere in detail.

---

Dr. Robert Koch, the distinguished German bacteriologist, who is on a visit to this country, was tendered a dinner by the German Medical Society of the State of New York at the Waldorf-Astoria, Saturday evening, April 11, 1908. The president of the society, Dr. Carl Beck, presided at the dinner which was attended by about 450 admirers of the guest of honor. It was a most interesting occasion and the speech of Professor Koch, though short, was a gem in good taste and in simple but charming rhetoric.

---

Dr. Harvey W. Wiley, of Washington, D. C., chief chemist of the United States department of agriculture, was tendered a dinner in commemoration of the twenty-fifth anniversary of his service, at the Hotel Astor, New York, Thursday evening, April 9, 1908. Dr. William Jay Schieffelin presided at the dinner which was attended by about 200 chemists of New York and vicinity. On the following evening, April 10, the chemists of the city of Washington also tendered Dr. Wiley a banquet for the same reason.

## Figures and Tables

**Figure f1:**